# Vertical Stratification Increases the Capacity of Morphological Traits to Predict Trophic Position in Neotropical Ants

**DOI:** 10.1002/ece3.71850

**Published:** 2025-07-23

**Authors:** Jésica Vieira, Karen C. Neves, Lino A. Zuanon, Heloise Gibb, Alan N. Andersen, Heraldo L. Vasconcelos

**Affiliations:** ^1^ Instituto de Biologia, Universidade Federal de Uberlândia Uberlândia Minas Gerais Brazil; ^2^ School of Life and Environmental Sciences, Deakin University Burwood Victoria Australia; ^3^ School of Biological, Earth and Environmental Sciences, UNSW Kensington New South Wales Australia; ^4^ Research Institute for the Environment and Livelihoods, Charles Darwin University Darwin Northern Territory Australia

**Keywords:** feeding ecology, functional traits, predictive models, stable isotopes of nitrogen, tropical savannas, vertical stratification

## Abstract

Morphology is a key functional trait that influences the ecophysiology of organisms. The use of morphological traits for understanding functional ecology is common in studies of ants, especially relating to their feeding biology. However, there is limited information on the predictive value of these traits in identifying the trophic position of ants. Moreover, although vertical stratification is a common feature of many ant assemblages, with distinct arboreal and ground‐active communities, the influence of verticality on the relationship between morphology and trophic position is unknown. We used data from 73 ant species from Brazilian Cerrado to examine the strength of associations between nine morphological traits and trophic position, and to evaluate if this varies between vertical strata. No individual morphological trait explained variation in the trophic position of arboreal species. However, femur length, petiole length, and head width were each individually correlated with the trophic position of ground species, but only weakly so. Predictive capacity increased substantially when data from different traits were combined in multiple regression models, especially when separate equations were derived for the arboreal and ground faunas. The estimation error of these models was below 15% for 70% of the species, with the most informative traits being femur length, petiole length, and head width for ground species and eye length, mandible length, and Weber's length for arboreal species. These results indicate that the use of multiple morphological traits is an effective approach for predicting the trophic position of most ants in our Neotropical system, the efficacy of which is enhanced when the different vertical strata are taken into consideration. We believe that our approach to assessing the predictive capacity of morphological traits, through the use of multiple traits and consideration of different microhabitats, has wide applicability to functional studies not just of ants but to fauna more generally.

## Graphical abstract

We use data from 73 Neotropical ant species to examine the strength of associations between nine morphological traits and trophic position. No individual morphological trait explained variation in the trophic position of arboreal species, whereas three traits individually correlated with the trophic position of ground species, but only weakly so. Predictive capacity increased substantially when data from different traits were combined in multiple regression models, especially when separate equations were derived for the arboreal and ground ant faunas.

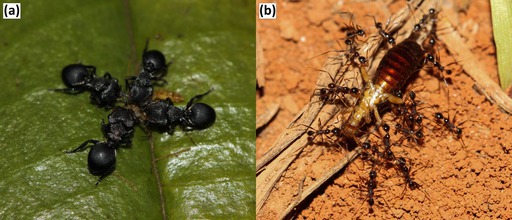

## 1 Introduction

Increasingly, ecologists are applying a functional‐trait approach to better understand the ecological roles of species and how biological communities are organized (Mouillot et al. 2013; Brousseau et al. 2018). Functional traits can relate to either species responses to environmental conditions (*response* traits) or to their effects on their physical or biotic environment (*effects* traits; Violle et al. 2007). Morphological traits can be either response or effect traits (or possibly both), but their functional significance is not yet well established for a range of organisms, notably invertebrates, whose natural history is poorly known (Brousseau et al. 2018).

Ants are a globally dominant faunal group, and morphological traits are commonly used in functional analyses of ant communities and their roles in ecosystems (e.g., Blaimer et al. 2015; Martello et al. 2018; Neves et al. 2024). For example, it has long been established that the ability of foraging ants to harvest resources is associated with their morphology, which can facilitate or constrain their ability to perform specific tasks such as prey capture (Wainwright 1994). The use of morphological traits as proxies in functional studies is especially valuable when detailed information on the biology of species is lacking, as is typically the case for ants (Weiser and Kaspari 2006; Gibb et al. 2015; Sosiak and Barden 2021; Drager et al. 2023). However, morphological traits can only be reliable proxies of ecological function if they have strong predictive capacity. Despite this, most studies of the association between morphological traits and ecological function in ants focus just on whether or not relationships are statistically significant. Moreover, in some cases, contrasting results have been reported; for instance, both positive (Gibb et al. 2015) and negative (Weiser and Kaspari 2006; Hanisch et al. 2020; Drager et al. 2023) relationships between ant body size and trophic position have been found. Therefore, there is still limited information on the predictive value of these traits, alone or in combination, in identifying the trophic position of ants (but see Drager et al. 2023). In addition, although vertical stratification is a common feature of many ant assemblages, the influence of verticality on the relationship between morphology and trophic position is unknown.

Ants are a particularly useful taxon for the study of the relationship between morphology and diet (Weiser and Kaspari 2006; Sosiak and Barden 2021; Drager et al. 2023). Most ants are generalist scavengers and predators of other arthropods, but many have highly specialized diets. At one end of the spectrum are species that specialize on seeds or nectar and therefore are essentially herbivorous; at the other end are species that specialize on live and often taxonomically restricted prey (Blüthgen and Feldhaar 2010). Furthermore, in the Americas, there is a diverse lineage of specialist ant fungivores (Mueller et al. 2001). Liquid carbohydrates, in the form of plant nectar or insect exudates, are a critically important food resource for most ants foraging in woody vegetation (Davidson et al. 2003). The ant faunas of many tropical habitats are highly stratified vertically, with little or no overlap between species foraging on the ground or in trees (Brühl et al. 1998; Vasconcelos and Vilhena 2006; Leahy et al. 2021). Vertical stratification is most characteristic of tropical rainforests, but it is also prominent in Brazilian savanna (*Cerrado*), where most arboreal species not only forage, but also nest in trees (Vasconcelos et al. 2023). Arboreal and ground‐dwelling ants in such highly stratified communities, therefore, tend to occupy different trophic positions (Vieira et al. 2021). In ants, there are significant phylogenetic signals in both trophic position (Drager et al. 2023) and vertical stratification (Lucky et al. 2013; Blaimer et al. 2015). Given that morphological traits also have a phylogenetic signal (Drager et al. 2023), one might therefore suspect that the relationship between morphological traits and trophic position differs between ground and arboreal ant communities. However, this is yet to be investigated.

Stable isotopic analyses have strongly improved our knowledge about the feeding ecology of ants (Davidson et al. 2003; Blüthgen et al. 2003; Fiedler et al. 2007; Kaspari et al. 2012; Gibb and Cunnigham 2011; Pfeiffer et al. 2014; Vieira et al. 2021). Once the relative proportion of the heavy ^15^N to light ^14^N isotopes (δ^15^N) in the body mass of different ant species is known, one can estimate the trophic position of these species within their communities (Feldhaar et al. 2010). In this study, we evaluate the extent to which morphological variation among Neotropical ant species can predict their trophic positions and how this varies between ground‐dwelling and arboreal assemblages. We achieve this based on the estimation of the trophic position of more than 70 species from the Brazilian Cerrado and the measurement of nine morphological traits assumed to be of relevance for the feeding ecology of ants (Parr et al. 2017).

We begin by describing the morphological differences between species that nest (or forage predominantly) in different vertical strata. We then examine the associations between individual morphological traits and trophic position and evaluate whether these associations vary between arboreal and ground‐dwelling species, with the expectation that, on average, arboreal species would occupy lower trophic positions. Since species traits are a product of phylogenetic history (Felsenstein 1985), we also test the influence of phylogeny on the relationship between morphological traits and trophic position. In addition, using multivariate regression, we evaluate the models that best explain the observed variation in trophic position among ant species based on the collective morphological traits we measured. Finally, we evaluate the predictive capacity of the multiple regression models based on morphological data in estimating the trophic position of Cerrado ants. In doing so, we examine the stratum specificity of models, testing our prediction that separate equations developed for ground‐dwelling and arboreal species perform better than a single equation that combines data from both strata.

## 2 Materials and Methods

### 2.1 Study Area and Sampling

Most of the trophic (isotopic) data analyzed here derives from two previous studies (Vieira et al. 2021; Zuanon et al. 2024) performed at the Panga Ecological Reserve (PER) located in Uberlândia, Minas Gerais, Brazil (19°10′S, 48°24′W), at ca. 800 m a.s.l. Additional unpublished data derives from ant sampling at the Santa Bárbara Ecological Station (SBES), which is in Águas de Santa Bárbara, São Paulo, Brazil (22°44′S, 49°13′ W), at approximately 650 m of elevation. At PER ants were collected in a savanna woodland and in the adjacent semideciduous forest, both on the ground and in trees, using a combination of pitfall trapping, Winkler samples, and active hand sampling (Vieira et al. 2021; Zuanon et al. 2024). At SESB ground‐dwelling ants were collected in a savanna woodland area using pitfall traps. Sampled ants from both sites were preserved in ethanol (92.8°) and sent for analysis of stable isotopes of nitrogen (δ^15^N) within 6 months after collection. Vegetation samples from all sites and sampled habitats were also sent for stable isotope analysis. Vegetation samples consisted of 20–30 leaves collected haphazardly along the ant‐sampling transects. Leaves collected in the same transect were pooled into a composite sample, mixed, and finely milled.

**FIGURE 1:**
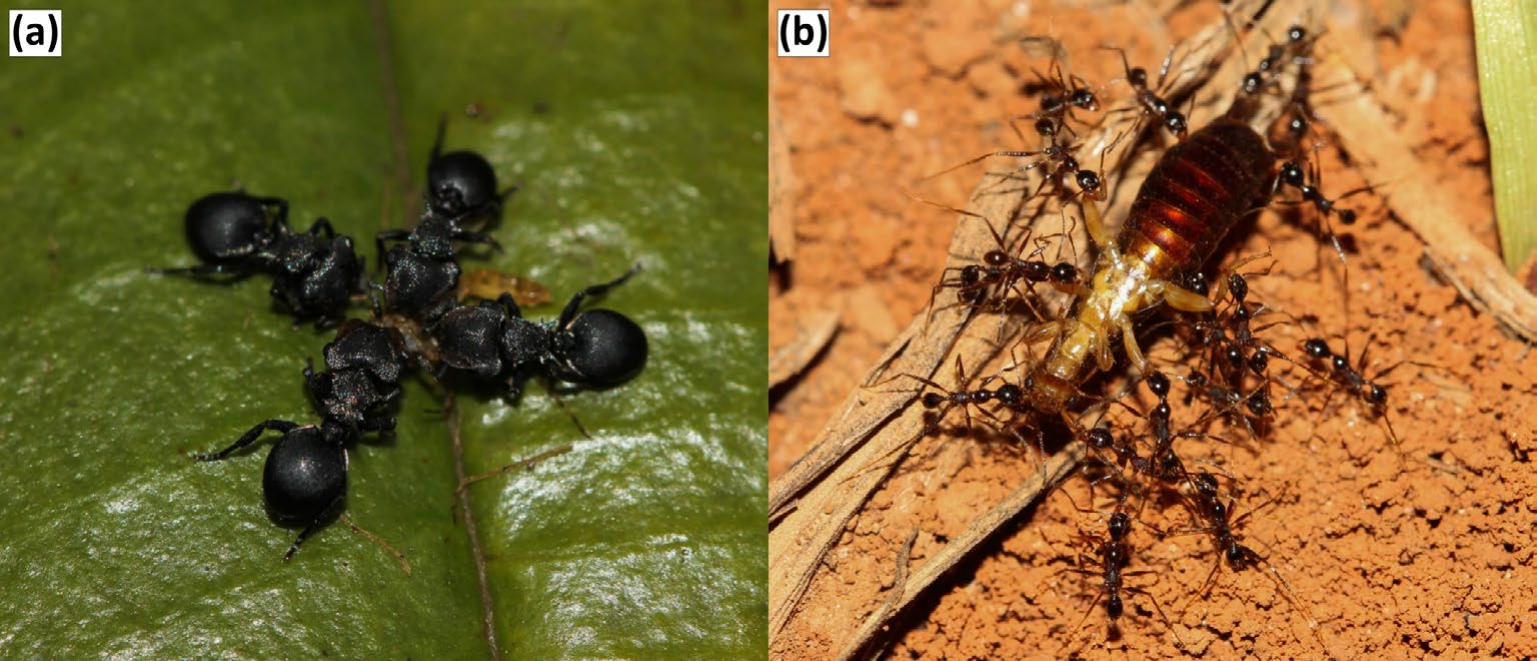
Neotropical ant species associated with different vertical strata. (a) Arboreal *Cephalotes pusillus* and (b) the ground‐dweller *Pheidole oxyops* . Photos credit: Adilson Quero Jr.

In total, 236 samples from 73 ant species (belonging to 30 genera) were sent for analysis of stable isotopes of nitrogen, comprising 43 ground‐dwelling species (from 24 genera) and 30 arboreal species (from 11 genera; Table S1). The stratum association of each ant species (Figure 1) was based, whenever possible, on well‐known specialization (e.g., species of *Cephalotes*, *Pseudomymrex*, and *Azteca* as arboreal specialists) and direct observations during sampling. In addition, we used previously published information about the Cerrado ant fauna (Neves et al. 2024), which considered both the relative frequency that each species was collected in ground vs. arboreal traps during previous ant surveys (Vasconcelos et al. 2023) and natural history data available at the Antweb platform (www.antweb.org). Except for *Ectatomma tuberculatum* , which nests on the ground but forages extensively in trees, and some *Camponotus* species that build polydomic nests both in trees and on ground ( *C. blandus* , *C. melanoticus* , *C.senex*, *and C. renggeri*), and forage in both strata, all species classified as arboreal nest in trees.

Isotopic data from 204 ant samples and seven composite vegetation samples (four from savanna and three from the adjacent semideciduous forest) were obtained at PER (Vieira et al. 2021; Zuanon et al. 2024), whereas data for 32 ant samples and eight vegetation samples were gathered at SBES. An ant sample consisted of individuals collected in the same sampling point (i.e., a tree, a 1 m^2^ sample of leaf‐litter or a 2.5 × 2.5 m grid with four pitfall traps each) and thus presumably belonging to the same colony. The number of samples per ant species ranged from one to 11 (mean = 3.22), depending both on species relative abundances and size of ant workers (given the difficulties in achieving the minimum mass required for isotopic analyses), and mean values are here reported (Table S1).

We removed the gaster of each worker during sample preparation to avoid the effect of recently ingested food items on the analysis (Blüthgen et al. 2003). Ant and vegetation samples were dried in an oven at 60°C for 48 h and then crushed with an agate mortar and pestle. The dried samples were put into small tin capsules in precisely weighed amounts (1.25–1.5 mg) then molded into a spherical shape, put on ELISE dishes, and sent to the University of California Stable Isotope Facility in Davis, California, USA. Results were expressed in delta (δ) notation per thousand, with an internationally acknowledged standard (atmospheric N_2_) as reference. The equation for the isotopic signatures is defined as δ^15^N (‰) = (Rsample − Rstandard)/Rstandard × 1.000, with R representing the molar ratio of the heavy/light isotope of the samples, and the atmospheric air is the standard used for nitrogen (Rstandard = 0.0036765).

Trophic position was determined using the following equation: TP = *λ* + (δ^15^N_ants_ − δ^15^N_vegetation_)/ΔN, where *λ* = 1 (the trophic level of autotrophs); ΔN = 3.4‰, which is the standard enrichment per trophic level (Post et al. Post 2002); δ^15^N_ants_ = the average value of δ^15^N obtained for a given ant species, and δ^15^N_vegetation_ = the average value of δ^15^ based on leaf samples taken within the area/habitat where the species was sampled.

### 2.2 Morphological Measurements

For each species we measured nine morphological traits, comprising: (a) Weber's length (a proxy of body size), (b) clypeus length, (c) eye length, (d) eye position, (e) femur length, (f) head width (above the eyes) (g) mandible length, (h) petiole length, and (i) scape length. A description of each trait and its potential in influencing the diet, foraging, and/or habitat use of ants is presented in Table S2. Measurements of pinned specimens were taken using an ocular micrometer mounted on a Leica M80 stereomicroscope. At least five ant workers from each species were measured, and average values were calculated for the measurements of each trait; when fewer than five workers were available, we measured all available specimens. For species with worker dimorphism or polymorphism, only minor workers were measured. Given the strong correlation of the measured morphological traits with body size, we standardized each trait (except Weber's length) by dividing the absolute value of the trait by Weber's length, and all the analyses were performed using the standardized values.

### 2.3 Phylogenetic Signal

Analysis of phylogenetic signal was based on species‐level phylogeny built with DNA sequences of ultraconserved elements of 357 Cerrado ant species (Neves et al. 2024). Prior to conducting the analysis, the phylogenetic tree was pruned to include only the 73 ant species relevant to the present study. We evaluated the degree of phylogenetic signal for trophic position and the nine morphological traits using Blomberg's *K*‐statistic (Blomberg et al. 2003). This metric uses a Brownian motion model of trait evolution to evaluate whether the observed distribution of trait values differs from expectation. A *K*‐value of less than one implies that related taxa resemble each other less than expected under a Brownian motion model of evolution, and a *K*‐value close to 0 indicates the random expectation (i.e., no phylogenetic signal). A *K*‐value greater than one implies that close relatives are more similar to each other than expected under Brownian motion (Blomberg et al. 2003). Analyses were performed using data from all species and then using data from the arboreal and ground‐dwelling species separately. The statistical significance of observed *K*‐values was assessed through randomization tests that produced a null distribution of 999 *K*‐values. We used the R package “phytools” (Revell 2012) to compute phylogenetic signals and statistical significance.

### 2.4 Data Analysis

We used Principal Component Analysis (PCA) to visualize the differences between the arboreal and ground‐dwelling species based on the nine morphological traits measured. All trait values were standardized (mean = 0, and SD = 1) prior to analysis, which was conducted in PcOrd 7.08 (McCune and Mefford 2018). A linear model using Phylogenetic Generalized Least Squares (PGLS; Freckleton et al. 2002) was built to evaluate the mean difference between species from different vertical strata in relation to: (a) trophic position and (b) each morphological trait. In the latter case, given the large number of tests performed (i.e., nine tests), which increases the chances of type I error, the resulting *p*‐values were adjusted using the Benjamin‐Hochberg method (Benjamini and Hochberg 1995). A PGLS model was used because it controls for the potential phylogenetic non‐independence of the model's residuals, whenever this is the case (Freckleton et al. 2002).

Similarly, we used PGLS models to analyze the capacity of each morphological trait in predicting trophic position. As above, the resulting *p*‐values were adjusted using the Benjamin‐Hochberg method (Benjamini and Hochberg 1995). The relationship between the individual traits and trophic position was also evaluated using Ordinary Least Squares (OLS) simple regressions. By comparing results from the OLS and PGLS models, we were able to determine the extent to which evolutionary history influences the relationship between morphological and dietary traits. PGLS uses a measure of phylogenetic correlation (lambda) as an index for measuring whether the data exhibit phylogenetic dependence or not (Freckleton et al. 2002). Lambda varies from zero to one, and the greater the value, the greater the degree of phylogenetic dependence in the data matrix. Because *λ* is estimated using residual errors (and not the original data) it can achieve a value of zero even when the predictor and the responsible variables show a phylogenetic signal. When *λ* equals zero (i.e., when the model's residuals are phylogenetic independent) results from the OLS and the PGLS models are equivalent (Pearse et al. 2023).

Finally, we performed multiple regression analyses (using both OLS and PGLS) to examine the capacity of combinations of morphological traits to predict trophic position. We checked for multicollinearity among the predictors using the Variance Inflation Factor (VIF) and found that after removing scape length from the data set, all remaining traits presented a VIF value < 2, which is indicative of no collinearity among predictors (Zuur et al. 2010). A stepwise procedure was used for model selection. Starting with the full model (i.e., with the model containing all the eight predictor morphological variables) we removed, one by one, the variable with the largest *p*‐value until all variables remaining in the model presented a *p* < 0.15. The adequacy of the resulting multiple regression models in predicting the trophic position (TP) of each species was evaluated using the percentage prediction error (PPE), defined as the difference between the estimated and observed values, expressed as a percentage. For this, we used the formula: PPE = [observed TP − estimated TP]/observed TP × 100 (Churchill et al. 2014).

Statistical analyses were conducted in R (R Core Team 2022) using the species‐level phylogeny described above and the packages “ape” (Paradis and Schliep 2019), “caper” (Orme et al. 2018), “geiger” (Pennell et al. 2014), and “pls” (Mevik and Wehrens 2007). All R scripts are available in Supporting Information.

## 3 Results

### 3.1 Differences in Ant Morphology and Trophic Position Between Vertical Strata

**FIGURE 2:**
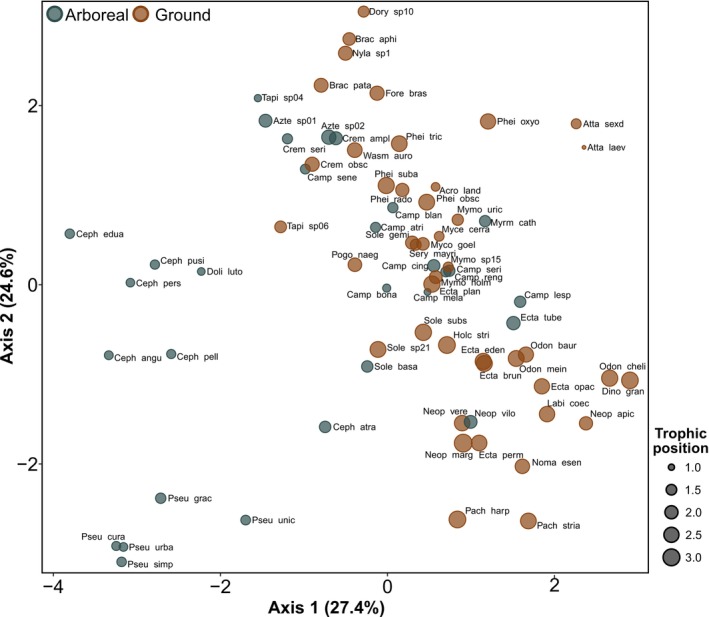
Principal component analysis (PCA) ordination plot of 73 Neotropical ant species based on nine morphological traits. Symbol size is proportional to the trophic position of each species. The full species names can be found in Table S1.

The PCA ordination of the 73 species studied according to the nine morphological traits showed high overlap among ground and arboreal species but that both strata included distinctive morphologies (Figure 2). For the arboreal assemblage, this distinction was primarily for species of *Cephalotes* and *Pseudomyrmex*, whereas for the ground assemblage, it involved many genera (Figure 2). On average, arboreal species had significantly larger eyes (relative to body size) and eyes positioned more dorsally, whereas the mean femur and mandible lengths were all significantly greater in the ground‐dwelling species (PGLS linear model, Adjusted *p* < 0.04 in all comparisons; Figure S1). We did not find significant differences between arboreal and ground‐dwelling species for head width, clypeus length, scape length, petiole length, and Weber's length (Figure S1).

Trophic position varied nearly four‐fold, from 0.93 in fungus‐growing *Atta laevigata* to 3.34 in the predator *Neoponera marginata*, with a mean of 1.95 (±0.63, SD). Mean trophic position was 1.54 times greater among the ground‐dwelling than among the arboreal species (arboreal = 1.49 ± 0.31; ground‐dwelling 2.29 ± 0.57; PGLS: *F*_1,71_ = 32.9, *p* < 0.001).

### 3.2 Phylogenetic Signal

Blomberg's *K*‐statistic values for trophic position ranged from 0.45 (considering only the arboreal species) to 0.63 (considering all species) (Table 1). *K*‐values between 0.4 and 0.7 are often regarded as indicative of a moderate phylogenetic signal, whereas those below 0.4 or higher than 0.7 are representatives of weak and strong phylogenetic signals, respectively (cf. Blaimer et al. 2015). Of the nine morphological traits evaluated in the analyses involving all 73 ant species, six displayed strong phylogenetic signals, while the remaining three (eye position, femur length and petiole length) presented moderate signals (Table 1). Considering only the ground‐dwelling species, five traits displayed strong phylogenetic signals, while three (eye position, femur length, and head width) had moderate signals, and one (mandible length) a weak phylogenetic signal. For the arboreal species, both eye position and petiole length had no significant phylogenetic signal (i.e., *K* was not significantly different from zero). Both femur and Weber's length had a moderate phylogenetic signal, while the remaining traits of arboreal species all presented *K*‐values > 0.7 (Table 1).

**TABLE 1:** Phylogenetic signal (Blomberg's *K*‐statistic) for trophic position and for nine morphological ant traits.

Trait	All species		Only arboreal species		Only ground species	
	*K*‐statistic	*p*	*K*‐statistic	*p*	*K*‐statistic	*p*
Trophic position	0.63	0.001	0.45	0.022	0.59	0.001
Clypeus length	0.97	0.001	0.96	0.002	1.00	0.001
Eye length	1.44	0.001	1.34	0.001	1.14	0.001
Eye position	0.49	0.001	0.43	0.053	0.70	0.001
Femur length	0.44	0.001	0.54	0.01	0.55	0.001
Head width	0.84	0.001	1.04	0.001	0.69	0.001
Mandible Length	0.86	0.001	0.88	0.001	0.36	0.015
Scape length	0.92	0.001	1.07	0.001	0.80	0.001
Petiole length	0.66	0.001	0.40	0.113	1.01	0.001
Weber's length	0.82	0.001	0.52	0.005	1.28	0.001

*Note:* *p*‐values were adjusted using the Benjamin–Hochberg method.

### 3.3 Trophic Position and Individual Morphological Traits

**FIGURE 3:**
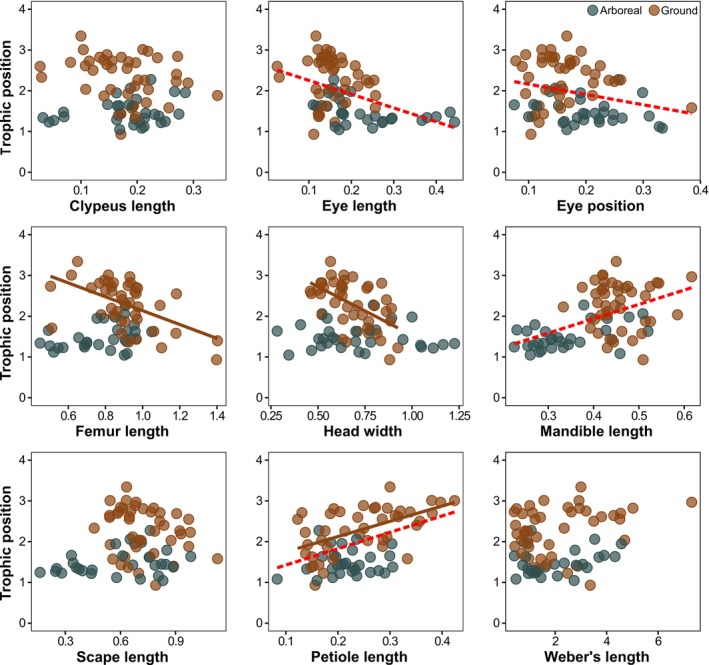
Scatterplot of the relationship between different morphological traits and the trophic position of arboreal and ground‐dwelling species. Analyses were performed separately for ants that forage predominantly on the ground or in the arboreal vegetation. Regression lines are depicted only for relationships that were significant according to Phylogenetic Generalized Least Squares (PGLS) regressions and presenting an adjusted *p*‐value < 0.05. Dashed red lines represent regression lines derived from models using data from all species, whereas continuous brown lines represent those derived from data from ground‐dwelling species only. All traits, except Weber's length (in mm), are relativized in relation to body size (Weber's length).

**FIGURE 4:**
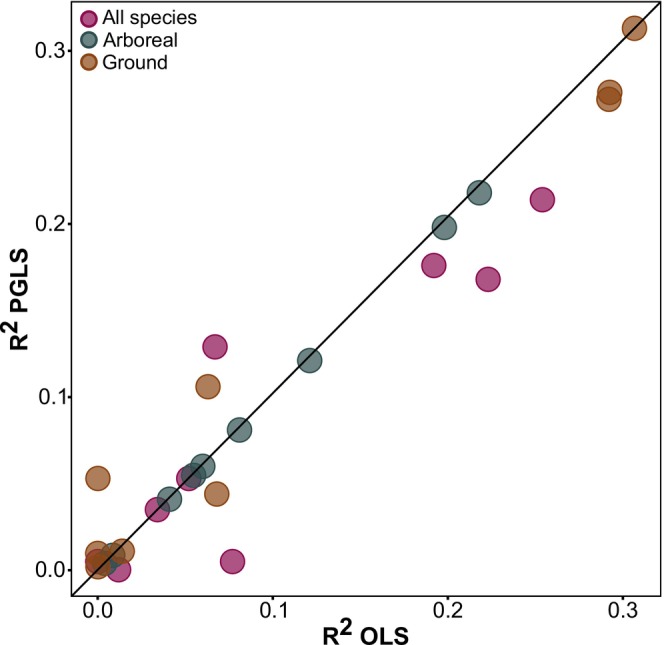
Coefficient of determination (*R* ^2^) of the OLS and PGLS regression models looking at the relationship between trophic position and individual morphological traits. Symbols with different colors represent models built with information from all species, the ground‐dwelling, or the arboreal species only. The continuous line represents the equivalence line, indicating that the OLS and PGLS models provided the same results and therefore the absence of phylogenetic dependence in the data.

In the analysis involving all the 73 ant species, we found that trophic position was positively related to mandible (*r* ^2^ = 0.21) and petiole lengths (*r* ^2^ = 0.17) and negatively related to both eye length (*r* ^2^ = 0.18) and eye position (*r* ^2^ = 0.13) (PGLS simple regressions; Adjusted *p* < 0.004 in all analyses; Figure 3, Table S3). In the analyses involving only the arboreal species, trophic position was not significantly related to any of the morphological traits (Adjusted *p* > 0.063 in all analyses), whereas in those with just the ground‐dwelling species, trophic position was negatively related to femur length (*r* ^2^ = 0.22) and head width (*r* ^2^ = 0.28), and was positively related to petiole length (*r* ^2^ = 0.31) (Adjusted *p* < 0.008 in all analyses; Figure 3, Table S3). Accounting for phylogeny had little or no influence on the amount of variation explained by the regression models, especially those involving only the arboreal species (Figure 4).

### 3.4 Estimation of Trophic Position Based on Multiple Morphological Traits

In the PGLS analysis involving all ant species, our stepwise procedure selected mandible length, petiole length, head width, and eye position as the most important multiple correlates of trophic position (Adjusted *r* ^2^ of the full model = 0.32). The first two morphological traits were positively related, whereas the latter two were negatively related to trophic position (Table 2). The stepwise OLS regression model also selected mandible length, petiole length, and head width as correlates of trophic position, and in addition, Weber's length and eye length (Adjusted *r* ^2^ of the full model = 0.47; Table 2).

**TABLE 2:** Results of the stepwise multiple regression analysis evaluating the influence of nine morphological traits on trophic position in the analyses involving all ant species, only the arboreal, or only the ground‐dwellers.

Trait	All species (*n* = 73)		Ground‐dwellers (*n* = 43)		Arboreal (*n* = 30)	
	OLS	PGLS	OLS	PGLS	OLS	PGLS
Intercept	1.337 (0.413)	1.804 (0.438)	3.878 (0.538)	3.878 (0.538)	0.962 (0.315)	0.962 (0.315)
Eye length	−1.946 (0.786)				−0.881 (0.542)	−0.881 (0.542)
Eye Position		−1.268 (0.741)				
Femur length			−1.062 (0.357)	−1.062 (0.357)		
Head width	−0.818 (0.337)	−0.789 (0.311)	−1.792 (0.478)	−1.792 (0.478)		
Mandible length	2.342 (0.699)	1.111 (0.696)			1.744 (0.612)	1.744 (0.612)
Petiole length	3.599 (0.833)	2.309 (0.608)	3.180 (0.864)	3.180 (0.864)		
Weber's length	−0.082 (0.047)				0.072 (0.042)	0.072 (0.042)
Lambda	NA	0.742	NA	0	NA	0
Adjusted *R* ^2^	0.467	0.325	0.544		0.335	0.335
PPE (mean ± SD)	21.5% ± 18.7%	25.1% ± 19.3%	14.8% ± 12.5%	14.8% ± 12.5%	11.8% ± 11.6%	11.8% ± 11.6%

*Note:* For each set of data, two regression models were run, one accounting for the evolutionary history of the species (phylogenetic generalized least squares—PGLS) and one not accounting (ordinary least squares—OLS). Predictor variables were selected using a stepwise procedure (in which predictors with the lowest *F* values and an *α* > 0.15 were removed manually from the model). Shown are the intercept, regression coefficients (with standard errors within parentheses), amount of variation explained (*R* ^2^), and the percentage prediction error (PPE) of each model. PGLS lambda values indicate the degree of phylogenetic dependence of the data.

Abbreviation: NA, not applicable.

Results from the PGLS and OLS models were identical for the analyses involving the arboreal or the ground‐dwelling species only. The most important correlates of trophic position for the arboreal species were mandible length, Weber's length, and eye length (Adjusted *r* ^2^ = 0.34), whereas for the ground‐dwelling species, the predictors selected were head width, femur length, and petiole length (Adjusted *r* ^2^ = 0.54; Table 2).

**FIGURE 5:**
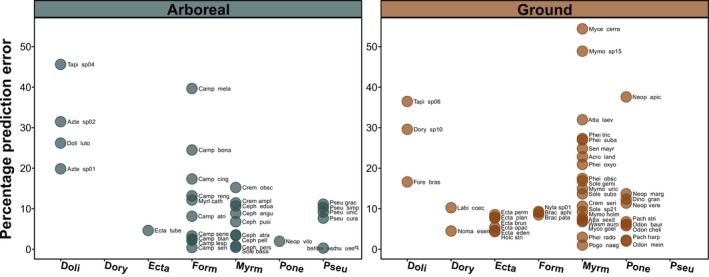
Percentage prediction error of the multiple regression models in estimating the trophic position of arboreal and ground‐dwelling species belonging to different ant subfamilies. Doli, Dolichoderinae; Dory, Dorylinae; Ecta, Ectatomminae; Form, Formicinae; Myrm, Myrmicinae; Ponde, Ponerinae; Pseu, Pseudomyrmecinae.

The mean error (PPE) in estimating trophic position using the above‐described models was 1.6 (OLS) and 1.9 (PGLS) times higher when using a single model (with data from all species) than when using separate ground and arboreal models (paired *t*‐tests, OLS model *t* = 4.13, df = 72, *p* < 0.001; PGLS model *t* = 5.35, df = 72, *p* < 0.001; Table 2). Thus, for the purpose of estimating the trophic position of ants based on their morphology, we considered only the separate models. Despite the differences in *r* ^2^ values (Table 2), we did not detect any significant difference in the percentage estimation error (PPE) between strata (*t*‐test; *t* = 1.03, df = 71, *p* = 0.305). For most (70%) species within both faunas, PPE values were below 15%; however, PPE values above 40% were also detected in a few cases (Figure 5). PPE values were consistently high (> 15%) for dolichoderines, especially for the two species of *Tapinoma*. Conversely, PPE values were consistently low (< 10%) for Ectatommines, including the six species of *Ectatomma*. The highest PPE values were shown by the fungus‐growing species *Mycetagroicus cerradensis* (54.4%) and *Mycetomoellerius* sp. 15 (48.8%). PPE values were sometimes highly variable within a genus, such as for *Camponotus* (0.41% to 39.6%) and *Pheidole* (2.9% to 27.3%) (Figure 5).

## 4 Discussion

Morphological traits are often associated with the functional ecology of species, including the ecological roles they play (Luck et al. 2012) and their responses to environmental change (e.g., Kimball et al. 2016). However, in many cases, the capacity of morphological traits to predict ecological function has been inadequately tested. Here we evaluated the capacity of morphological traits to predict trophic position in a vertically stratified Neotropical ant assemblage.

### 4.1 Differences in Ant Morphology and Trophic Position Between Vertical Strata

Trophic structure differs substantially between ground and arboreal ant assemblages, and so our first aim was to evaluate the extent to which species from different vertical strata differ in their morphology. PCA revealed substantial morphological overlap, but both strata had highly distinctive elements in arboreal species of *Cephalotes* and *Pseudomyrmex* and ground‐nesting poneromorphs. We found that, on average, arboreal species have eyes that are 1.5 times longer and 1.26 times more dorsally positioned than do ground species, whereas the latter have mandibles that are 2.1 times longer. These findings are consistent with those of a previous study at the Cerrado–Amazon transition (Almeida et al. 2023). However, in contrast to the latter study, we did not find that arboreal species are larger (i.e., have a greater Weber's length), but we found that the relative length of the femur of ground species is 16% larger on average than those that live in trees. Lack of differences in mean body size between ground and arboreal species was also noted in a study in the forests of Panama (Yanoviak and Kaspari 2000). These somewhat different findings point to regional differences in assemblage morphology due to differences in species composition.

Larger and more dorsally positioned eyes are potentially an advantage for arboreal species because they rely more on visual navigation than those that forage on the ground (Almeida et al. 2023). Similarly, smaller legs are of advantage for ants foraging in more complex habitats, and this may well also be the case in the three‐dimensional canopy habitat of arboreal ants (Farji‐Brener et al. 2004). Differences in mandible length between species from different strata, as found here and elsewhere (Almeida et al. 2023), likely reflect the differences in food resources, given that many arboreal species have a predominantly liquid diet of plant and insect exudates, whereas those on the ground rely more on resources derived from detritivore (“brown” rather than “green”) food webs (Yanoviak and Kaspari 2000; Davidson et al. 2003). Indeed, relatively short mandibles were a characteristic of the most “herbivorous” arboreal species, notably those in the genera *Camponotus*, *Cephalotes*, and *Pseudomyrmex* (Table S1).

It is important to note that our study did not consider very small species because we could not obtain enough mass for isotopic analysis. We therefore did not consider the morphologically distinctive cryptobiotic fauna of soil and litter, which is characterized by small body sizes, short appendages, and tiny or absent eyes, and includes a range of morphologically unique taxa such as the highly diverse genus *Strumigenys*. Many of these species feed on small invertebrate prey and thus occupy a high trophic position (Mezger and Pfeiffer 2010). Therefore, the relationships between ant morphology and trophic position we describe here can potentially be altered as data from cryptobiotic species become available, especially given that most of the predatory species we analyzed were large‐sized poneromorphs.

As expected, mean trophic position differed strongly between arboreal and ground species. In the Neotropics, genus‐level composition is very different in trees compared with on the ground (Yanoviak and Kaspari 2000; Almeida et al. 2023; Vieira et al. 2021; Vasconcelos et al. 2023). However, we found substantial variation in trophic position within a genus, related to where they forage. For example, arboreal foraging *Ectatomma tuberculatum* occupies a lower trophic position than all the other (ground‐foraging) *Ectatomma* species, as does arboreal *Solenopsis* sp. nr. *basalis* compared with the other (ground‐dwelling) species of *Solenopsis*. We suggest that these differences reflect food‐web differences in the isotopic signature of ant invertebrate prey. Although all species of *Ectatomma* and *Solenopsis* are regarded as omnivore/predatory (Brown 2000), both *E. tuberculatum* and *S*. nr. *basalis*—in contrast to their relatives—have a stronger link with green rather than brown food webs (Vieira et al. 2021). In green food webs primary consumers are herbivores, while in brown food webs the primary consumers are invertebrate detritivores. As the latter consume not only plant detritus but also bacteria and fungi (which themselves are also primary consumers), their δ^15^N is higher than that of invertebrates associated with green food webs (Steffan et al. 2017). This consequently reflects in the isotopic signature of ants that feed on invertebrates. In other words, closely related species associated with different vertical strata may be functionally equivalent but show differences in trophic position (derived from δ^15^N measurements) due to differences in the isotopic signature of their prey.

### 4.2 Trophic Position and Individual Morphological Traits

Previous studies investigating the potential association between morphological traits and trophic position in ants have often found statistically significant relationships (Weiser and Kaspari 2006; Gibb et al. 2015; Drager et al. 2023). However, variation in these relationships according to nesting/foraging stratum has not previously been investigated. We found that the relationships between morphological traits and trophic position vary markedly between arboreal and ground ant species. Femur length, petiole length, and head width were all associated with trophic position for ground species but not for arboreal species. Further, there were several instances in which a given trait was not correlated with trophic position for species within a stratum but was significant when considering all species. This was the case for the relationship between relative mandible length and trophic position, where the overall relationship can be explained by arboreal species having lower trophic levels and shorter mandibles and the reverse being true for ground‐dwelling species. Similarly, the overall negative relationship between relative eye length and trophic position largely resulted from differences in eye length between the arboreal and ground‐dwelling species. In this sense, our findings may help to reconcile some of the contrasting results observed in previous studies. For instance, studies on ground ant communities in Australia have reported a positive relationship between eye width and trophic position (Gibb et al. 2015), whereas broader analyses incorporating data from multiple ant communities, including arboreal species, have found the opposite pattern—consistent with our findings (Drager et al. 2023).

Despite a consideration of vertical stratum leading to an improvement in understanding the relationships between morphology and trophic position, we found that the strength of the relationships was relatively weak, typically explaining < 30% of the variance. This reinforces previous findings that individual morphological traits have low predictive power in estimating trophic position (Drager et al. 2023). However, it is important to mention that, among ants, similarity in trophic position does not necessarily reflect similarity in diet, as any two morphologically distinct species may feed on different food items and still present a similar δ^15^N signature. This makes it particularly difficult for individual morphological traits to show a strong correlation with trophic position. For instance, our data show that *Mycocepurus goeldii* and *Brachymyrmex* sp. nr. *aphidicola* (both ground‐dwellers) occupy a similar trophic position, even though these two species have highly distinct diets (fungus‐culturing vs. honeydew specialist respectively) and morphologies.

Interestingly, accounting for phylogeny had relatively little influence on relationships between individual morphological traits and trophic position (cf. Gibb et al. 2015). Phylogenetic correction had no effect for arboreal species, suggesting that recent morphological trait divergences among arboreal species are likely to be as important as divergences deeper in the phylogeny (Gibb et al. 2015).

### 4.3 Estimation of Trophic Position Based on Multiple Morphological Traits

Our final aim was to evaluate the capacity of multiple regression models to predict the trophic position of Cerrado ants, and whether equations specific for species from each vertical stratum perform better than a single equation combining data from species from both strata. Studies with vertebrates have shown that multiple regression models perform better than simple linear regression models in predicting body mass based on cranial measurements (Churchill et al. 2014). Here, using a similar approach, we also found a much stronger relationships between the morphology of ant species and their trophic position when using multiple regression models. Since many of the ant genera and several of the species that were included in these models are widespread in the Neotropics, the potential of these models in helping to predict the trophic position of ants from regions other than the Cerrado can be high.

Notably, model accuracy increased when separate equations were derived for the arboreal and ground faunas. Prediction errors were relatively low (PPE < 15%), and consistently so for the arboreal genera *Cephalotes* and *Pseudomyrmex*, and also for ground‐foraging Ectatomminae and Ponerinae species. In contrast, the PPE of these models was relatively high for most dolichoderines (from both strata) and for several ground Myrmicinae, especially the fungus‐growing ants and species of *Pheidole*. The trophic position of most fungus‐growing ants was overestimated, whereas that of several *Pheidole* species was underestimated by the multiple regression model. Petiole length was a predictor of the trophic position of ground‐dwelling ants, and species of *Pheidole* have unusually short petioles. Similarly, both head width and femur length were negatively related to trophic position for ground species, and fungus‐growing ants have relatively long legs and large heads.

## 5 Conclusion

We have shown that the use of multiple morphological traits is an effective approach for predicting the trophic position of most ants in our Neotropical system, the efficacy of which is enhanced when the different vertical strata are taken into consideration. Moreover, we also found evidence that the capacity of morphological traits to predict trophic position can be highly taxon‐specific (cf. Drager et al. 2023), which enhances the broader applicability of our findings. For example, predictive capacity was high for all six species of *Ectatomma*, suggesting that this is characteristic of a genus that is prominent throughout the Neotropics. The reverse is the case for dolichoderines, especially species of *Tapinoma*. In this sense, future studies should focus on obtaining isotopic data for more species, especially those belonging to taxa for which our models presented a relatively poor fit or for which we were unable to obtain data (e.g., cryptobiotic species). The overall accuracy of our multiple regression models could also be improved by developing models that are specific to different ant subfamilies.

Our study has focused on a vertically stratified neotropical ant fauna. However, we believe that our approach to assessing the predictive capacity of morphological traits, through the use of multiple traits and consideration of different microhabitats, has wide applicability to functional studies not just of ants but to fauna more generally.

## Author Contributions

**Jésica Vieira:** conceptualization (equal), data curation (equal), formal analysis (equal), investigation (lead), methodology (equal), writing – original draft (supporting), writing – review and editing (equal). **Karen C. Neves:** data curation (equal), formal analysis (equal), writing – review and editing (equal). **Lino A. Zuanon:** data curation (equal), formal analysis (equal), writing – review and editing (equal). **Heloise Gibb:** conceptualization (equal), methodology (equal), writing – review and editing (equal). **Alan N. Andersen:** conceptualization (equal), writing – original draft (supporting), writing – review and editing (equal). **Heraldo L. Vasconcelos:** conceptualization (equal), formal analysis (equal), funding acquisition (equal), investigation (equal), supervision (equal), writing – original draft (lead), writing – review and editing (equal).

## Conflicts of Interest

The authors declare no conflicts of interest.

## Supporting information


Data S1.

## Data Availability

All the required data are uploaded as Supporting Information.
