# Supplementary material for: Vertical Stratification Increases the Capacity of Morphological Traits to Predict Trophic Position in Neotropical Ants
Source: Ecol Evol. 2025 Jul 23;15(7):e71850. doi: 10.1002/ece3.71850 (PMC12286619; doi:10.1002/ece3.71850)
Supplement: Supplementary file 1 — Data S1. [file ECE3-15-e71850-s001.docx]

**Supplementary information**

Table S1. List of the ant species, their nesting/foraging stratum, trophic position (TP), relative clypeus length (CL), relative eye length (EL), eye position (EP), relative femur length (FL), relative head width across eyes (HAE), relative mandible length (ML), relative petiole length (PL), relative scape length (SL) and Weber´s length (WL).

| Species code | Species name | stratum | TP | CL | EL | EP | FL | HAE | ML | PL | SL | WL |
| --- | --- | --- | --- | --- | --- | --- | --- | --- | --- | --- | --- | --- |
| Acro land | *Acromyrmex landolti* | ground | 1.224 | 0.204 | 0.161 | 0.118 | 1.102 | 0.925 | 0.430 | 0.172 | 0.699 | 2.480 |
| Atta laev | *Atta laevigata* | ground | 0.931 | 0.171 | 0.112 | 0.104 | 1.398 | 0.881 | 0.509 | 0.156 | 0.792 | 3.363 |
| Atta sexd | *Atta sexdens* | ground | 1.398 | 0.167 | 0.125 | 0.111 | 1.403 | 0.833 | 0.486 | 0.153 | 0.889 | 2.880 |
| Azte sp02 | *Azteca sp. 2* | arb | 2.275 | 0.224 | 0.159 | 0.257 | 0.911 | 0.794 | 0.379 | 0.164 | 0.762 | 1.070 |
| Azte sp01 | *Azteca sp.1* | arb | 1.954 | 0.286 | 0.170 | 0.299 | 0.791 | 0.891 | 0.394 | 0.208 | 0.677 | 0.939 |
| Brac aphi | *Brachymyrmex nr. aphidicola* | ground | 1.883 | 0.343 | 0.257 | 0.211 | 0.886 | 0.886 | 0.514 | 0.286 | 0.971 | 0.438 |
| Brac pata | *Brachymyrmex patagonicus* | ground | 2.191 | 0.290 | 0.242 | 0.213 | 0.832 | 0.890 | 0.460 | 0.242 | 0.929 | 0.517 |
| Camp atri | *Camponotus atriceps* | arb | 1.421 | 0.231 | 0.182 | 0.248 | 0.906 | 0.596 | 0.306 | 0.241 | 0.788 | 2.817 |
| Camp blan | *Camponotus blandus* | arb | 1.448 | 0.215 | 0.177 | 0.216 | 0.965 | 0.529 | 0.309 | 0.206 | 0.823 | 1.969 |
| Camp bona | *Camponotus bonariensis* | arb | 1.159 | 0.194 | 0.187 | 0.180 | 0.828 | 0.466 | 0.285 | 0.236 | 0.807 | 2.088 |
| Camp cing | *Camponotus cingulatus* | arb | 1.791 | 0.199 | 0.172 | 0.190 | 0.956 | 0.408 | 0.271 | 0.228 | 0.852 | 2.769 |
| Camp lesp | *Camponotus lespesii* | arb | 1.635 | 0.168 | 0.130 | 0.152 | 0.919 | 0.283 | 0.253 | 0.170 | 0.983 | 4.324 |
| Camp mela | *Camponotus melanoticus* | arb | 1.046 | 0.169 | 0.171 | 0.167 | 0.902 | 0.345 | 0.260 | 0.160 | 0.831 | 2.738 |
| Camp reng | *Camponotus renggeri* | arb | 1.444 | 0.171 | 0.179 | 0.195 | 0.935 | 0.504 | 0.350 | 0.211 | 0.846 | 3.075 |
| Camp sene | *Camponotus senex* | arb | 1.378 | 0.239 | 0.256 | 0.310 | 0.913 | 0.700 | 0.328 | 0.305 | 0.888 | 1.616 |
| Camp seri | *Camponotus sericeiventris* | arb | 1.620 | 0.192 | 0.143 | 0.232 | 1.049 | 0.559 | 0.294 | 0.262 | 0.756 | 3.699 |
| Ceph angu | *Cephalotes angulatus* | arb | 1.246 | 0.250 | 0.275 | 0.163 | 0.688 | 1.050 | 0.225 | 0.263 | 0.163 | 1.250 |
| Ceph atra | *Cephalotes atratus* | arb | 1.663 | 0.147 | 0.179 | 0.109 | 0.865 | 0.808 | 0.231 | 0.192 | 0.333 | 3.900 |
| Ceph edua | *Cephalotes eduarduli* | arb | 1.329 | 0.213 | 0.296 | 0.263 | 0.699 | 1.226 | 0.278 | 0.140 | 0.374 | 1.375 |
| Ceph pell | *Cephalotes pellans* | arb | 1.288 | 0.164 | 0.238 | 0.196 | 0.687 | 1.045 | 0.267 | 0.278 | 0.393 | 1.070 |
| Ceph pers | *Cephalotes persimilis* | arb | 1.218 | 0.242 | 0.273 | 0.160 | 0.545 | 1.113 | 0.307 | 0.203 | 0.442 | 0.963 |
| Ceph pusi | *Cephalotes pusillus* | arb | 1.304 | 0.196 | 0.294 | 0.223 | 0.764 | 1.166 | 0.345 | 0.233 | 0.446 | 1.233 |
| Crem ampl | *Crematogaster sp. nr. ampla* | arb | 1.980 | 0.266 | 0.199 | 0.137 | 0.851 | 1.000 | 0.478 | 0.186 | 0.714 | 0.805 |
| Crem obsc | *Crematogaster sp. nr. obscuricornis* | ground | 2.258 | 0.213 | 0.253 | 0.240 | 0.907 | 0.800 | 0.427 | 0.213 | 0.787 | 0.938 |
| Crem seri | *Crematogaster sp. nr. sericea* | arb | 1.427 | 0.274 | 0.220 | 0.199 | 0.844 | 0.952 | 0.441 | 0.199 | 0.667 | 0.744 |
| Dino gran | *Dinoponera grandis* | ground | 2.968 | 0.219 | 0.151 | 0.192 | 0.932 | 0.685 | 0.616 | 0.397 | 0.685 | 7.300 |
| Doli luto | *Dolichoderus lutosus* | arb | 1.119 | 0.212 | 0.162 | 0.328 | 0.522 | 0.718 | 0.281 | 0.190 | 0.579 | 1.448 |
| Dory sp10 | *Dorymyrmex sp.10* | ground | 1.581 | 0.256 | 0.256 | 0.385 | 1.179 | 0.769 | 0.410 | 0.333 | 1.128 | 0.975 |
| Ecta brun | *Ectatomma brunneum* | ground | 2.752 | 0.141 | 0.156 | 0.172 | 0.922 | 0.625 | 0.453 | 0.320 | 0.656 | 3.200 |
| Ecta eden | *Ectatomma edentatum* | ground | 2.821 | 0.172 | 0.131 | 0.141 | 0.843 | 0.521 | 0.441 | 0.270 | 0.614 | 2.944 |
| Ecta opac | *Ectatomma opaciventris* | ground | 2.555 | 0.155 | 0.135 | 0.154 | 0.965 | 0.465 | 0.425 | 0.285 | 0.648 | 4.512 |
| Ecta perm | *Ectatomma permagnum* | ground | 2.642 | 0.146 | 0.149 | 0.173 | 0.801 | 0.497 | 0.418 | 0.310 | 0.541 | 4.275 |
| Ecta plan | *Ectatomma planidens* | ground | 2.841 | 0.272 | 0.152 | 0.136 | 0.816 | 0.584 | 0.424 | 0.272 | 0.600 | 2.500 |
| Ecta tube | *Ectatomma tuberculatum* | arb | 2.060 | 0.188 | 0.156 | 0.146 | 0.903 | 0.521 | 0.509 | 0.236 | 0.653 | 3.535 |
| Fore bras | *Forelius brasiliensis* | ground | 2.265 | 0.201 | 0.235 | 0.261 | 1.094 | 0.710 | 0.448 | 0.175 | 0.923 | 0.844 |
| Holc stri | *Holcoponera striatula* | ground | 3.011 | 0.210 | 0.141 | 0.139 | 0.816 | 0.571 | 0.420 | 0.380 | 0.632 | 1.563 |
| Labi coec | *Labidus coecus* | ground | 2.598 | 0.027 | 0.025 | 0.089 | 0.959 | 0.618 | 0.484 | 0.238 | 0.541 | 1.428 |
| Myce cerra | *Mycetagroicus cerradensis* | ground | 1.367 | 0.197 | 0.123 | 0.106 | 0.925 | 0.650 | 0.444 | 0.148 | 0.641 | 1.250 |
| Mymo holm | *Mycetomoellerius holmgreni* | ground | 1.928 | 0.183 | 0.124 | 0.095 | 0.970 | 0.633 | 0.402 | 0.142 | 0.544 | 1.760 |
| Mymo sp15 | *Mycetomoellerius sp. 15* | ground | 1.433 | 0.171 | 0.122 | 0.126 | 0.966 | 0.632 | 0.436 | 0.164 | 0.603 | 1.663 |
| Mymo uric | *Mycetomoellerius urichii* | ground | 1.627 | 0.194 | 0.140 | 0.118 | 1.060 | 0.710 | 0.462 | 0.151 | 0.624 | 1.860 |
| Myco goel | *Mycocepurus goeldii* | ground | 1.871 | 0.141 | 0.160 | 0.154 | 0.904 | 0.769 | 0.526 | 0.179 | 0.686 | 0.975 |
| Myrm cath | *Myrmelachista catharinae* | arb | 1.629 | 0.165 | 0.117 | 0.114 | 0.960 | 0.701 | 0.520 | 0.196 | 0.760 | 0.810 |
| Neop apic | *Neoponera apicalis* | ground | 2.035 | 0.109 | 0.117 | 0.160 | 0.777 | 0.521 | 0.585 | 0.287 | 0.702 | 4.700 |
| Neop marg | *Neoponera marginata* | ground | 3.343 | 0.100 | 0.117 | 0.167 | 0.650 | 0.567 | 0.450 | 0.300 | 0.633 | 3.000 |
| Neop vere | *Neoponera verenae* | ground | 2.612 | 0.108 | 0.209 | 0.225 | 0.834 | 0.459 | 0.454 | 0.316 | 0.649 | 3.800 |
| Neop vilo | *Neoponera villosa* | arb | 1.948 | 0.163 | 0.147 | 0.191 | 0.755 | 0.549 | 0.430 | 0.304 | 0.574 | 4.571 |
| Noma esen | *Nomamyrmex esenbeckii* | ground | 2.331 | 0.030 | 0.030 | 0.100 | 0.930 | 0.590 | 0.409 | 0.250 | 0.457 | 2.350 |
| Nyla sp1 | *Nylanderia sp.1* | ground | 2.228 | 0.270 | 0.186 | 0.250 | 0.975 | 0.657 | 0.333 | 0.137 | 0.980 | 0.816 |
| Odon baur | *Odontomachus bauri* | ground | 2.503 | 0.121 | 0.157 | 0.200 | 0.886 | 0.629 | 0.514 | 0.357 | 0.814 | 3.500 |
| Odon cheli | *Odontomachus chelifer* | ground | 2.823 | 0.133 | 0.131 | 0.145 | 0.934 | 0.516 | 0.540 | 0.295 | 0.781 | 5.020 |
| Odon mein | *Odontomachus meinerti* | ground | 2.730 | 0.115 | 0.129 | 0.180 | 0.834 | 0.626 | 0.525 | 0.339 | 0.766 | 2.630 |
| Pach harp | *Pachycondyla harpax* | ground | 3.008 | 0.104 | 0.121 | 0.123 | 0.617 | 0.638 | 0.421 | 0.424 | 0.544 | 2.925 |
| Pach stria | *Pachycondyla striatula* | ground | 2.696 | 0.081 | 0.123 | 0.123 | 0.725 | 0.630 | 0.469 | 0.381 | 0.584 | 4.540 |
| Phei obsc | *Pheidole obscuricornis* | ground | 2.710 | 0.185 | 0.155 | 0.143 | 0.964 | 0.530 | 0.375 | 0.131 | 0.815 | 1.120 |
| Phei oxyo | *Pheidole oxyops* | ground | 2.549 | 0.182 | 0.160 | 0.164 | 1.182 | 0.513 | 0.405 | 0.123 | 0.971 | 1.223 |
| Phei rado | *Pheidole radoszkowskii* | ground | 2.030 | 0.153 | 0.185 | 0.150 | 0.962 | 0.736 | 0.417 | 0.169 | 0.876 | 0.698 |
| Phei suba | *Pheidole subarmata* | ground | 2.809 | 0.236 | 0.176 | 0.124 | 0.768 | 0.839 | 0.544 | 0.192 | 0.724 | 0.500 |
| Phei tric | *Pheidole triconstricta* | ground | 2.688 | 0.198 | 0.179 | 0.173 | 0.973 | 0.760 | 0.422 | 0.185 | 0.906 | 0.731 |
| Pogo naeg | *Pogonomyrmex naegelli* | ground | 2.116 | 0.196 | 0.216 | 0.157 | 0.863 | 0.863 | 0.431 | 0.294 | 0.667 | 1.275 |
| Pseu cura | *Pseudomyrmex curacaensis* | arb | 1.271 | 0.052 | 0.367 | 0.247 | 0.502 | 0.518 | 0.255 | 0.227 | 0.247 | 1.255 |
| Pseu urba | *Pseudomyrmex gr. urbanus* | arb | 1.231 | 0.043 | 0.443 | 0.223 | 0.572 | 0.623 | 0.333 | 0.290 | 0.280 | 1.137 |
| Pseu grac | *Pseudomyrmex gracilis* | arb | 1.473 | 0.069 | 0.433 | 0.231 | 0.656 | 0.579 | 0.321 | 0.215 | 0.343 | 2.353 |
| Pseu simp | *Pseudomyrmex simplex* | arb | 1.339 | 0.033 | 0.378 | 0.185 | 0.448 | 0.550 | 0.290 | 0.189 | 0.253 | 0.935 |
| Pseu unic | *Pseudomyrmex unicolor* | arb | 1.362 | 0.070 | 0.400 | 0.100 | 0.700 | 0.780 | 0.370 | 0.230 | 0.360 | 3.242 |
| Sery mayri | *Sericomyrmex mayri* | ground | 1.573 | 0.190 | 0.121 | 0.156 | 0.887 | 0.814 | 0.506 | 0.190 | 0.558 | 1.444 |
| Sole gemi | *Solenopsis geminata* | ground | 2.028 | 0.220 | 0.147 | 0.130 | 0.845 | 0.690 | 0.430 | 0.257 | 0.713 | 0.906 |
| Sole basa | *Solenopsis sp. nr. basalis* | arb | 1.653 | 0.185 | 0.110 | 0.075 | 0.493 | 0.775 | 0.427 | 0.300 | 0.612 | 0.416 |
| Sole sp21 | *Solenopsis sp.21* | ground | 2.734 | 0.155 | 0.097 | 0.078 | 0.505 | 0.767 | 0.476 | 0.204 | 0.592 | 0.412 |
| Sole subs | *Solenopsis substituta* | ground | 2.885 | 0.146 | 0.138 | 0.095 | 0.794 | 0.640 | 0.403 | 0.249 | 0.680 | 1.012 |
| Tapi sp04 | *Tapinoma sp04* | arb | 1.082 | 0.208 | 0.250 | 0.333 | 0.917 | 0.750 | 0.458 | 0.083 | 0.708 | 0.480 |
| Tapi sp06 | *Tapinoma sp06* | ground | 1.708 | 0.216 | 0.187 | 0.171 | 0.512 | 0.764 | 0.393 | 0.138 | 0.782 | 0.692 |
| Wasm auro | *Wasmannia auropunctata* | ground | 2.403 | 0.270 | 0.216 | 0.216 | 0.891 | 0.864 | 0.459 | 0.351 | 0.837 | 0.463 |

Table S2. List of the nine morphological traits measured and their potential relationship with the diet, food searching behavior or habitat use by ants.

| **Morphological trait** | **Description of the measurement** | **Potential relationship with** **diet, food searching behavior or habitat use** | **Source** |
| --- | --- | --- | --- |
| Clypeus length (CL) |  | Indicative of sucking ability and liquid-feeding behavior | Parr et al. (2017) and references therein |
| Eye length (EL) | Maximum length of the eye | Indicative of foraging period, food-searching behavior and habitat type | Martello et al (2018) and references therein |
| Eye position (EP) | Head width across the eyes minus head width between the eyes. Higher eye position indicates more dorsal eyes | Eye position can influence the performance of predatory species | Gibb and Parr (2013) |
| Femur length (FL) | Length of the femur of the hind leg | Indicative of foraging speed and thermoregulatory strategy | Parr et al. (2017) and references therein |
| Head across eyes (HAE) | Maximum head width | Indicative of the size of spaces through which ant can pass and of mandibular musculature. (wider heads accommodate larger mandibular muscles that allow capture of larger or fiercer prey) | Martello et al. (2018) and references therein |
| Mandible length (ML) | Straight-line distance from the insertion to the tip of the mandible | Indicative of diet: longer mandibles = more predatory | Parr et al. (2017) and references therein |
| Petiole length (PL) | Maximum petiole length, not including postpetiole if present | Correlated to behavior of predatory species and their performance in prey capture | Martello et al (2018) and references therein |
| Scape length (SL) | Length of the antennal scape | Indicative of sensory abilities: longer scape facilitates the following of pheromone trails | Martello et al (2018) and references therein |
| Weber’s length (WL) | Distance from the anterodorsal margin of the pronotum to the posteroventral margin of the propodeum | Indicative of body size, which is related to prey size selection during solitary foraging microhabitats in which different species forage, metabolic characteristics and resource use | Martello et al. (2018) and references therein |

Table S3. Phylogenetic Generalized Least Squares (PGLS) simple linear regression models, evaluating the degree of relationship between each morphological trait and trophic position. Separate analyses were performed using data from all ant species (n = 73), from the arboreal (n = 30), or from the ground-dwelling species (n = 43). Show are the regression coefficients (and their standard errors), the amount of variation explained (R2) the degree of phylogenetic dependence of the data (λ). Significant relationships are highlighted in bold (Benjamin-Hochberg Adjusted P < 0.05).

| Trait | All species | | | Ground-dwellers | | | Arboreal | | |
| --- | --- | --- | --- | --- | --- | --- | --- | --- | --- |
|  | Coefficient (SE) | R^2^ | λ | Coefficient (SE) | R2 | λ | Coefficient (SE) | R2 | λ |
| Clypeus lenght | 0.178 (1.067) | 0.001 | 0.596 | -1.928(1.404) | 0.044 | 0.279 | 1.082 (0.848) | 0.055 | 0 |
| Eye lenght | **-3.167 (0.814)** | **0.176** | **0.542** | -1.175 (1.748) | 0.011 | 0.346 | -1.471 (0.558) | 0.198 | 0 |
| Eye Position | **-2.959 (0.911)** | **0.129** | **0.684** | -0.880 (1.536) | 0.002 | 0.343 | -0.958 (0.876) | 0.041 | 0 |
| Femur lenght | 0.222 (0.366) | 0.005 | 0.617 | **-1.683(0.430)** | **0.272** | **0.169** | 0.650(0.331) | 0.121 | 0 |
| Head across eyes | -0.224 (0.387) | 0.005 | 0.590 | **-2.439 (0.616)** | **0.276** | **0.107** | -0.082 (0.237) | 0.004 | 0 |
| Mandible lenght | **3.086 (0.700)** | **0.214** | **0.629** | 0.924 (1.455) | 0.009 | 0.355 | 1.756 (0.628) | 0.218 | 0 |
| Petiole lenght | **2.971 (0.784)** | **0.168** | **0.622** | **3.623 (0.841)** | **0.313** | **0.216** | 0.578 (1.168) | 0.009 | 0 |
| Scape lenght | 0.580 (0.359) | 0.035 | 0.598 | -0.880 (0.576) | 0.053 | 0.405 | 0.388 (0.246) | 0.081 | 0 |
| Weber´s lenght | 0.086 (0.043) | 0.053 | 0.660 | 0.114 (0.051) | 0.106 | 0.418 | 0.063 (0.047) | 0.060 | 0 |

Figure S1. Box plots showing the differences in morphological traits between arboreal and ground-dwelling species. All traits, except Weber´s length, were relativized in relation to Weber´s length. Different letters indicate significant differences between mean values (PGLS, Adjusted P < 0.05).


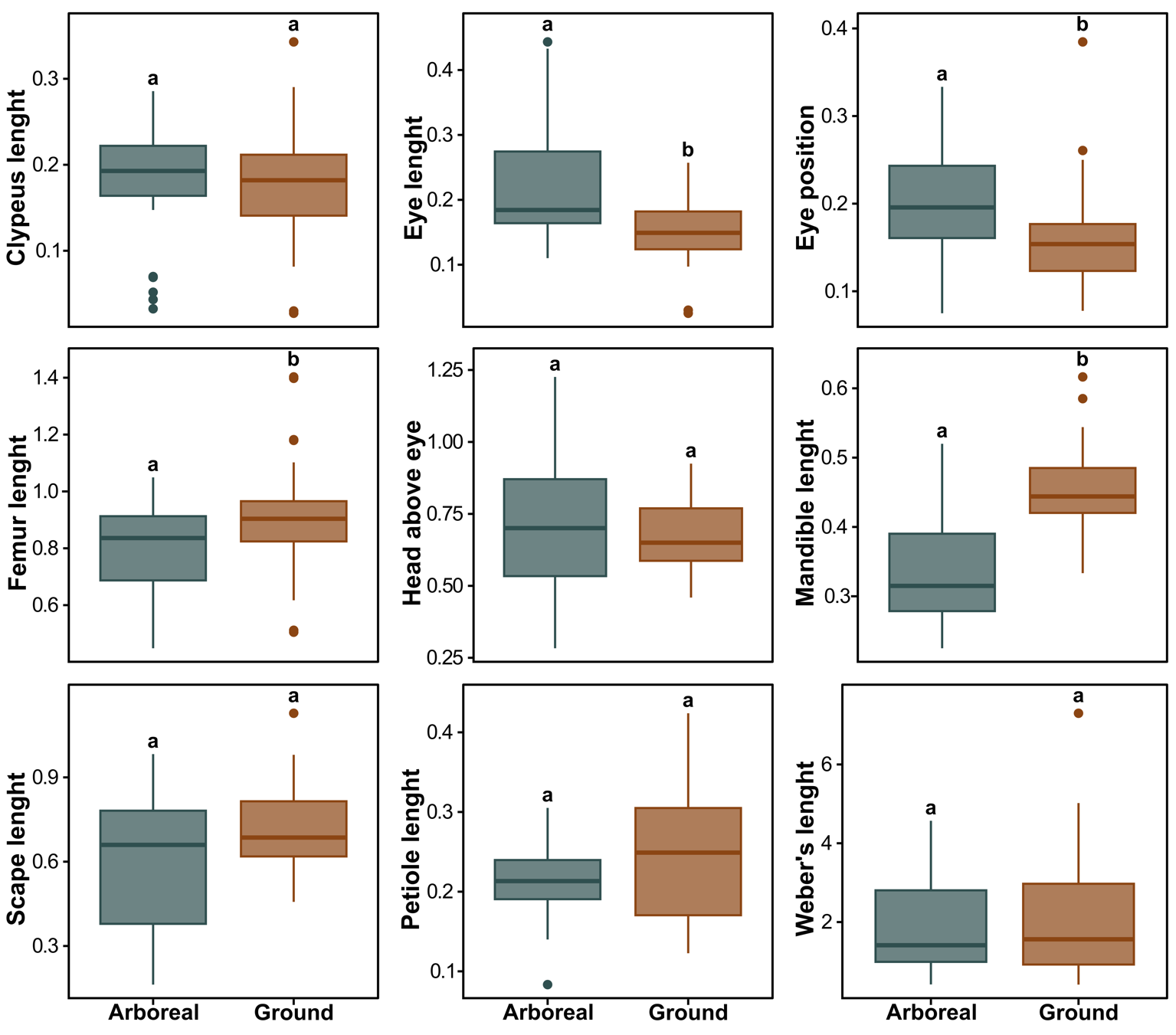


**R SCRIPTS**

#load packages

library(ape)

library(caper)

library(geiger)

library(picante)

library(nlme)

library(dplyr)

library(tidyverse)

library(car)

library(lme4)

library(lmerTest)

library(devtools)

library(DHARMa)

#####################

#STEP 1: DATA INPUT, CHECK LABELS, REMOVING UNWANTED TIPS, MAKE "comparative.data" OBJECT

# Using ALL SPECIES (arboreal and ground species)

#input phylogenetic tree

ant_tree<-read.tree("tree_file.phy") #.phy or .nex extensions are allowed

#dataset of ALL SPECIES

data_community<-read.table("comu.txt", header = T, row.names = 1) #example how to input .txt file. For .csv file need to use read.csv function. ##observations for this matrix: 1) species are rows and sites are columns; 2)encourage to include a column containing the labels that represents the ant species in the phylogenetic tree. 3)For comparison between arboreal and ground community, need to include a column with species strata preference (i.e.: arboreal; ground).

##trait data of ALL SPECIES

data_trait<-read.table("traits.txt", header = T, row.names=1) #example how to input .txt file. For .csv file need to use read.csv function #species are rows and traits are columns

##use phylogenetic tree labels as row names for data_community

rownames(data_community)<-data_community$name_tree #using the column with phylogenetic tree labels

##including only species presented in the data_community matrix

subset_ant_tree <- drop.tip(phy = ant_tree,

tip = setdiff(ant_tree$tip.label,

rownames(data_community)))

#Name check whether the names match in the data and the tree

check<-name.check(subset_ant_tree, data_community, data.names = subset_ant_tree$tip.label)

#run object check

check

#Make a column that includes the specie names to generate a "comparative.data" object:

data_community$species<-row.names(data_community)

# Make a "comparative.data" object using traits and phylogeny information:

species_traits<-comparative.data(phy=subset_ant_tree, data=data_community, names.col=species, vcv=TRUE)

#####################

###STEP 2: Phylogenetic generalized linear models between strata and each trait

####The results can be observed in the Figure S3 (Supplementary material).

##The "pgls" function is used to run this model. IMPORTANT: in this function, the lambda argument is using "ML" option. "ML" means that the model are considering: The strength and type of the phylogenetic signal in the data matrix (values range from 0 to 1. Meaning: 0 low signal and 1 high signal). More details are available in the "pgls" R Documentation.

##IMPORTANT: STEP 2 needs to be run for each trait used in the study.

model_strata<-pgls(data_community$petiole~data_community$stratum, data=species_traits, lambda = "ML")

summary(model_strata)

anova(model_strata)

#####################

###STEP 3: Phylogenetic generalized linear models between trophic position and each trait. The results can be observed in Table S3 (Supplementary material).

##The "pgls" function is used to run this model. IMPORTANT: in this function, the lambda argument is using "ML" option. "ML" means that the model are considering: The strength and type of the phylogenetic signal in the data matrix (values range from 0 to 1. Meaning: 0 low signal and 1 high signal). More details are available in the "pgls" R Documentation.

##Firstly, run the Phylogenetic generalized linear models using ALL SPECIES

###IMPORTANT: STEP 3 needs to be run for each trait used in the study.

model_trophic<-pgls(data_community$posi_trofica~data_community$Eye_position, data=species_traits, lambda = "ML")

summary(model_trophic)

#################

#STEP 3.A: Run the Phylogenetic generalized lineal models using only ARBOREAL SPECIES

#Before running model, is important to filter the arboreal species in the complete data set and phylogenetic tree

#filtering the arboreal ants from data_community matrix

data_arb<-data_community[data_community$stratum=="arb",]

#Make a column that includes the specie names to generate a "comparative.data" object:

data_arb$species<-row.names(data_arb)

##including only species presented in the data_arb matrix

arb_ant_tree <- drop.tip(phy = ant_tree,

tip = setdiff(ant_tree$tip.label,

rownames(data_arb)))

# Make a "comparative.data" object using traits and phylogeny information:

species_traits_arb<-comparative.data(phy=arb_ant_tree, data=data_arb, names.col=species, vcv=TRUE)

#Run the Phylogenetic generalized lineal models using only ARBOREAL SPECIES. Observe argument lambda is using "ML".

###IMPORTANT: The Phylogenetic generalized lineal models needs to be run for each trait used in the study.

attach(data_arb)

model_trophic.A<-pgls(data_arb$posi_trofica~data_arb$Eye_position, data=species_traits_arb, lambda = "ML")

summary(model_trophic.A)

#####################

#STEP 3.B: Run the Phylogenetic generalized lineal models using only GROUND SPECIES. #Before running model, is important to filter the ground species in the complete data set and phylogenetic tree.

data_ground<-data_community[data_community$stratum=="ground",]

#Make a column that includes the specie names to generate a "comparative.data" object:

data_ground$species<-row.names(data_ground)

##remove unwanted species name

grd_ant_tree <- drop.tip(phy = ant_tree,

tip = setdiff(ant_tree$tip.label,

rownames(data_ground)))

# Make a "comparative.data" object using traits and phylogeny information:

species_traits_solo<-comparative.data(phy=grd_ant_tree, data=data_ground, names.col=species, vcv=TRUE)

#Run the Phylogenetic generalized lineal models using only GROUND SPECIES. Observe argument lambda is using "ML".

###IMPORTANT: The Phylogenetic generalized lineal models needs to be run for each trait used in the study.

attach(data_ground)

model_trophic.B<-pgls(data_ground$posi_trofica~data_ground$weber, data=species_traits_solo, lambda = "ML")

summary(model_trophic.B)

#####################

###STEP 4

#######STEPWISE REGRESSION ##############

###The results can be observed in Table 2.

#IMPORTANT: The variables were removed one by one. It started removing the variable with largest P-value until all variables remaining in the model presented a P < 0.15.

#USING ALL SPECIES

library(usdm) #load package

#test for multicollineariy using vif function

attach(data_community)

vif(data_community[,c(9,10,12,13,15,17,18,22)]) #selecting the columns to be tested (VIF < 2)

###STEPWISE REGRESSION using pgls function

model1<-pgls(data_community$posi_trofica ~ data_community$weber + data_community$Hae + data_community$cly_r + data_community$mandible + data_community$eye + data_community$femur + data_community$petiole + data_community$Eye_position, data=species_traits, lambda = "ML")

summary(model1)

#remove Weber's length

model2<-pgls(data_community$posi_trofica ~ data_community$Hae + data_community$cly_r + data_community$mandible + data_community$eye + data_community$femur + data_community$petiole + data_community$Eye_position, data=species_traits, lambda = "ML")

summary(model2)

#remove Eye length

model3<-pgls(data_community$posi_trofica ~ data_community$Hae+data_community$cly_r + data_community$mandible + data_community$femur + data_community$petiole + data_community$Eye_position, data=species_traits, lambda = "ML")

summary(model3)

#remove Clypeus length

model4<-pgls(data_community$posi_trofica ~ data_community$Hae + data_community$mandible + data_community$femur + data_community$petiole + data_community$Eye_position, data=species_traits, lambda = "ML")

summary(model4)

#remove Femur length

model_final<-pgls(data_community$posi_trofica ~ data_community$Hae + data_community$mandible + data_community$petiole + data_community$Eye_position, data=species_traits, lambda = "ML")

summary(model_final)

#Stepwise is done.

#to get model diagnostic plots for PGLS

par (mfrow = c(2,2))

plot(model_final)

#likelihood profiles

par (mfrow = c(1,1))

lambda.trait<-pgls.profile(model_final,"lambda")

plot(lambda.trait)

#####STEPWISE REGRESSION using "lm" function

lm_model1<-lm(data_community$posi_trofica ~ data_community$weber + data_community$Hae + data_community$cly_r + data_community$mandible + data_community$eye + data_community$femur + data_community$petiole + data_community$Eye_position, data=data_community)

summary(lm_model1)

# remove clypeus length

lm_model2<-lm(data_community$posi_trofica ~ data_community$weber + data_community$Hae + data_community$mandible + data_community$eye + data_community$femur + data_community$petiole + data_community$Eye_position, data=data_community)

summary(lm_model2)

# remove Eye position

lm_model3<-lm(data_community$posi_trofica ~ data_community$weber + data_community$Hae + data_community$mandible + data_community$eye+data_community$femur + data_community$petiole, data=data_community)

summary(lm_model3)

#remove Femur length femur

lm_model_final<-lm(data_community$posi_trofica ~ data_community$weber + data_community$Hae + data_community$mandible + data_community$eye + data_community$petiole, data=data_community)

summary(lm_model_final)

#to get model diagnostic plots for "lm"

par (mfrow = c(2,2))

plot(lm_model_final)

############################################

##### Stepwise pgls using ARBOREAL ANTS ONLY

model_arb1<-pgls(data_arb$posi_trofica ~ data_arb$weber + data_arb$Hae + data_arb$cly_r + data_arb$mandible + data_arb$eye + data_arb$femur + data_arb$petiole + data_arb$Eye_position, data=species_traits_arb, lambda = "ML")

summary(model_arb1)

#remove Eye position

model_arb2<-pgls(data_arb$posi_trofica ~ data_arb$Hae + data_arb$cly_r + data_arb$mandible + data_arb$eye + data_arb$femur + data_arb$petiole + data_arb$weber, data=species_traits_arb, lambda = "ML")

summary(model_arb2)

#remove femur length

model_arb3<-pgls(data_arb$posi_trofica ~ data_arb$Hae + data_arb$cly_r + data_arb$mandible + data_arb$eye + data_arb$petiole + data_arb$weber, data=species_traits_arb, lambda = "ML")

summary(model_arb3)

#remove clypeus length

model_arb4<-pgls(data_arb$posi_trofica ~ data_arb$Hae + data_arb$mandible + data_arb$eye + data_arb$petiole + data_arb$weber, data=species_traits_arb, lambda = "ML")

summary(model_arb4)

#remove petiole length

model_arb5<-pgls(data_arb$posi_trofica ~ data_arb$Hae + data_arb$mandible + data_arb$eye + data_arb$weber, data=species_traits_arb, lambda = "ML")

summary(model_arb5)

#remove Head above eye

model_arb_final<-pgls(data_arb$posi_trofica ~ data_arb$mandible + data_arb$eye + data_arb$weber, data=species_traits_arb, lambda = "ML")

summary(model_arb_final)

##stepwise model is done

#to get model diagnostic plots for PGLS

par (mfrow = c(2,2))

plot(model_arb_final)

#likelihood profiles

par (mfrow = c(1,1))

lambda.trait<-pgls.profile(model_arb_final,"lambda")

plot(lambda.trait)

##################################

#####Stepwise LM ARBOREAL ANTS ONLY

lm_arb1<-lm(Posicao_trofica ~ weber + Hae + cly_r + mandible + eye + femur + petiole + spine + Eye_position, data = data_arb)

summary(lm_arb1)

#remove petiole length

lm_arb2<-lm(Posicao_trofica ~ weber + Hae + cly_r + mandible + eye + femur + spine + Eye_position, data = data_arb)

summary(lm_arb2)

# remove eye position

lm_arb3<-lm(Posicao_trofica ~ weber + Hae + cly_r + mandible + eye + femur + spine, data = data_arb)

summary(lm_arb3)

# remove femur length

lm_arb4<-lm(Posicao_trofica ~ weber + Hae + cly_r + mandible + eye + spine, data = data_arb)

summary(lm_arb4)

# remove spine

lm_arb5<-lm(Posicao_trofica ~ weber + Hae + cly_r + mandible + eye, data = data_arb)

summary(lm_arb5)

#remove cly_r

lm_arb6<-lm(Posicao_trofica ~ weber + Hae + mandible + eye, data = data_arb)

summary(lm_arb6)

# remove eye

lm_arb_final<-lm(Posicao_trofica~weber+Hae+mandible, data = data_arb)

summary(lm_arb_final)

#Stepwise lm is done.

#to get model diagnostic plots for "lm"

par (mfrow = c(2,2))

plot(lm_arb_final)

##########################################

##### Stepwise pgls using GROUND ANTS ONLY

model_grd1<-pgls(data_ground$posi_trofica ~ data_ground$weber + data_ground$Hae + data_ground$cly_r + data_ground$mandible + data_ground$eye + data_ground$femur + data_ground$petiole + data_ground$Eye_position, data=species_traits_solo, lambda = "ML")

summary(model_grd1)

# remove clypeus length

model_grd2<-pgls(data_ground$posi_trofica ~ data_ground$weber + data_ground$Hae + data_ground$mandible + data_ground$eye + data_ground$femur + data_ground$petiole + data_ground$Eye_position, data=species_traits_solo, lambda = "ML")

summary(model_grd2)

#remove mandible length

model_grd3<-pgls(data_ground$posi_trofica ~ data_ground$weber + data_ground$Hae + data_ground$eye + data_ground$femur + data_ground$petiole + data_ground$Eye_position, data=species_traits_solo, lambda = "ML")

summary(model_grd3)

#remove eye length

model_grd4<-pgls(data_ground$posi_trofica ~ data_ground$weber + data_ground$Hae + data_ground$femur + data_ground$petiole + data_ground$Eye_position, data=species_traits_solo, lambda = "ML")

summary(model_grd4)

# remove Eye position

model_grd5<-pgls(data_ground$posi_trofica ~ data_ground$weber + data_ground$Hae + data_ground$femur + data_ground$petiole, data=species_traits_solo, lambda = "ML")

summary(model_grd4)

#remove Weber's length

model_grd_final<-pgls(data_ground$posi_trofica ~ data_ground$Hae + data_ground$femur + data_ground$petiole, data=species_traits_solo, lambda = "ML")

summary(model_grd_final)

##stepwise model is done

#to get model diagnostic plots for PGLS

par (mfrow = c(2,2))

plot(model_grd_final)

#likelihood profiles

par (mfrow = c(1,1))

lambda.trait<-pgls.profile(model_grd_final,"lambda")

plot(lambda.trait)

##################################

#####Stepwise LM GROUND ANTS ONLY

lm_ground1<-lm(Posicao_trofica ~ weber + Hae + cly_r + mandible + eye + femur + petiole + spine + Eye_position, data = data_ground)

summary(lm_ground1)

#remove Clypeus length

lm_ground2<-lm(Posicao_trofica ~ weber + Hae + mandible + eye + femur + petiole + spine + Eye_position, data = data_ground)

summary(lm_ground2)

#remove Mandible length

lm_ground3<-lm(Posicao_trofica ~ weber + Hae + eye + femur + petiole + spine + Eye_position, data = data_ground)

summary(lm_ground3)

#remove Weber's length

lm_ground4<-lm(Posicao_trofica ~ Hae + eye + femur + petiole + spine + Eye_position, data = data_ground)

summary(lm_ground4)

#remove Eye length

lm_ground5<-lm(Posicao_trofica ~ Hae + femur + petiole + spine + Eye_position, data = data_ground)

summary(lm_ground5)

#remove Eye position

lm_ground6<-lm(Posicao_trofica ~ Hae + femur + petiole + spine, data = data_ground)

summary(lm_ground6)

#remove Spine

lm_ground_final<-lm(Posicao_trofica ~ Hae + femur + petiole, data = data_ground)

summary(lm_ground_final)

###Stepwise lm is done

#to get model diagnostic plots for "lm"

par (mfrow = c(2,2))

plot(lm_ground_final)

#####################

###STEP 5

##############################################

####Simple Regression Model

#The simple models were performed to show the Phylogeny influence on the amount of variation explained by the regression models. The results can be observed in Figure 3 (Main text).

##Simple Regression Model including ALL SPECIES

smodel1<-lm(Posicao_trofica~weber, data = data_community)

summary(smodel1)

sim_smodel1 <- simulateResiduals(smodel1, plot=T)

smodel2<-lm(Posicao_trofica~Hae, data = data_community)

summary(smodel2)

sim_smodel2 <- simulateResiduals(smodel2, plot=T)

smodel3<-lm(Posicao_trofica~Hah, data = data_community)

summary(smodel3)

sim_smodel3 <- simulateResiduals(smodel3, plot=T)

smodel4<-lm(Posicao_trofica~inter.oc, data = data_community)

summary(smodel4)

sim_smodel4 <- simulateResiduals(smodel4, plot=T)

smodel5<-lm(Posicao_trofica~cly_r, data = data_community)

summary(smodel5)

sim_smodel5 <- simulateResiduals(smodel5, plot=T)

smodel6<-lm(Posicao_trofica~mandible, data = data_community)

summary(smodel6)

sim_smodel6 <- simulateResiduals(smodel6, plot=T)

smodel7<-lm(Posicao_trofica~scapo, data = data_community)

summary(smodel7)

sim_smodel7 <- simulateResiduals(smodel7, plot=T)

smodel8<-lm(Posicao_trofica~eye, data = data_community)

summary(smodel8)

sim_smodel8 <- simulateResiduals(smodel8, plot=T)

smodel9<-lm(Posicao_trofica~femur, data = data_community)

summary(smodel9)

sim_smodel9 <- simulateResiduals(smodel9, plot=T)

smodel10<-lm(Posicao_trofica~petiole, data = data_community)

summary(smodel10)

sim_smodel10 <- simulateResiduals(smodel10, plot=T)

smodel11<-lm(Posicao_trofica~spine, data = data_community)

summary(smodel11)

sim_smodel11 <- simulateResiduals(smodel11, plot=T)

smodel12<-lm(Posicao_trofica~Eye_position, data = data_community)

summary(smodel12)

sim_smodel12 <- simulateResiduals(smodel12, plot=T)

#####################

##Simple Regression Model including GROUND SPECIES

s_grdmodel1<-lm(Posicao_trofica~weber, data = data_ground)

summary(s_grdmodel1)

sim_s_grdmodel1 <- simulateResiduals(s_grdmodel1, plot=T)

s_grdmodel2<-lm(Posicao_trofica~Hae, data = data_ground)

summary(s_grdmodel2)

sim_s_grdmodel2 <- simulateResiduals(s_grdmodel2, plot=T)

s_grdmodel3<-lm(Posicao_trofica~Hah, data = data_ground)

summary(s_grdmodel3)

sim_s_grdmodel3 <- simulateResiduals(s_grdmodel3, plot=T)

s_grdmodel4<-lm(Posicao_trofica~inter.oc, data = data_ground)

summary(s_grdmodel4)

sim_s_grdmodel4 <- simulateResiduals(s_grdmodel4, plot=T)

s_grdmodel5<-lm(Posicao_trofica~cly_r, data = data_ground)

summary(s_grdmodel5)

sim_s_grdmodel5 <- simulateResiduals(s_grdmodel5, plot=T)

s_grdmodel6<-lm(Posicao_trofica~mandible, data = data_ground)

summary(s_grdmodel6)

sim_s_grdmodel6 <- simulateResiduals(s_grdmodel6, plot=T)

s_grdmodel7<-lm(Posicao_trofica~scapo, data = data_ground)

summary(s_grdmodel7)

sim_s_grdmodel7 <- simulateResiduals(s_grdmodel7, plot=T)

s_grdmodel8<-lm(Posicao_trofica~eye, data = data_ground)

summary(s_grdmodel8)

sim_s_grdmodel8 <- simulateResiduals(s_grdmodel8, plot=T)

s_grdmodel9<-lm(Posicao_trofica~femur, data = data_ground)

summary(s_grdmodel9)

sim_s_grdmodel9 <- simulateResiduals(s_grdmodel9, plot=T)

s_grdmodel10<-lm(Posicao_trofica~petiole, data = data_ground)

summary(s_grdmodel10)

sim_s_grdmodel10 <- simulateResiduals(s_grdmodel10, plot=T)

s_grdmodel11<-lm(Posicao_trofica~spine, data = data_ground)

summary(s_grdmodel11)

sim_s_grdmodel11 <- simulateResiduals(s_grdmodel11, plot=T)

s_grdmodel12<-lm(Posicao_trofica~Eye_position, data = data_ground)

summary(s_grdmodel12)

sim_s_grdmodel12 <- simulateResiduals(s_grdmodel12, plot=T)

#####################

##Simple Regression Model including ARBOREAL SPECIES

s_arbmodel1<-lm(Posicao_trofica~weber, data = data_arb)

summary(s_arbmodel1)

sim_s_arbmodel1 <- simulateResiduals(s_arbmodel1, plot=T)

s_arbmodel2<-lm(Posicao_trofica~Hae, data = data_arb)

summary(s_arbmodel2)

sim_s_arbmodel2 <- simulateResiduals(s_arbmodel2, plot=T)

s_arbmodel3<-lm(Posicao_trofica~Hah, data = data_arb)

summary(s_arbmodel3)

sim_s_arbmodel3 <- simulateResiduals(s_arbmodel3, plot=T)

s_arbmodel4<-lm(Posicao_trofica~inter.oc, data = data_arb)

summary(s_arbmodel4)

sim_s_arbmodel4 <- simulateResiduals(s_arbmodel4, plot=T)

s_arbmodel5<-lm(Posicao_trofica~cly_r, data = data_arb)

summary(s_arbmodel5)

sim_s_arbmodel5 <- simulateResiduals(s_arbmodel5, plot=T)

s_arbmodel6<-lm(Posicao_trofica~mandible, data = data_arb)

summary(s_arbmodel6)

sim_s_arbmodel6 <- simulateResiduals(s_arbmodel6, plot=T)

s_arbmodel7<-lm(Posicao_trofica~scapo, data = data_arb)

summary(s_arbmodel7)

sim_s_arbmodel7 <- simulateResiduals(s_arbmodel7, plot=T)

s_arbmodel8<-lm(Posicao_trofica~eye, data = data_arb)

summary(s_arbmodel8)

sim_s_arbmodel8 <- simulateResiduals(s_arbmodel8, plot=T)

s_arbmodel9<-lm(Posicao_trofica~femur, data = data_arb)

summary(s_arbmodel9)

sim_s_arbmodel9 <- simulateResiduals(s_arbmodel9, plot=T)

s_arbmodel10<-lm(Posicao_trofica~petiole, data = data_arb)

summary(s_arbmodel10)

sim_s_arbmodel10 <- simulateResiduals(s_arbmodel10, plot=T)

s_arbmodel11<-lm(Posicao_trofica~spine, data = data_arb)

summary(s_arbmodel11)

sim_s_arbmodel11 <- simulateResiduals(s_arbmodel11, plot=T)

s_arbmodel12<-lm(Posicao_trofica~Eye_position, data = data_arb)

summary(s_arbmodel12)

sim_s_arbmodel12 <- simulateResiduals(s_grdmodel12, plot=T)
